# Microenvironment components and spatially resolved single-cell transcriptome atlas of breast cancer metastatic axillary lymph nodes: The lymph node atlas of breast cancer metastases

**DOI:** 10.3724/abbs.2022131

**Published:** 2022-09-21

**Authors:** Kun Xu, Runtian Wang, Qin Chen, Yiqiu Liu, Xintong Li, Ling Mao, Cenzhu Wang, Fangyan Gao, Longfei Hu, Hui Xie, Cong Wang, Guohua Zhou, Xiaoxiang Guan

**Affiliations:** 1 Department of Oncology the First Affiliated Hospital of Nanjing Medical University Nanjing 210029 China; 2 Singleron Biotechnologies Nanjing 210044 China; 3 Department of Breast Surgery the First Affiliated Hospital of Nanjing Medical University Nanjing 210029 China; 4 Department of Pathology the First Affiliated Hospital of Nanjing Medical University Nanjing 210029 China; 5 Department of Pharmacology Jinling Hospital Medical School of Nanjing University Nanjing 210002 China; 6 Jiangsu Key Lab of Cancer Biomarkers Prevention and Treatment Collaborative Innovation Center for Personalized Cancer Medicine Nanjing Medical University Nanjing 210003 China

**Keywords:** single-cell RNA sequencing, spatial transcriptomics, microenvironment, breast cancer, metastasis

## Abstract

As an indicator of clinical prognosis, lymph node metastasis of breast cancer has drawn great attention. Many reports have revealed the characteristics of metastatic breast cancer cells, however, the effect of breast cancer cells on the microenvironment components of lymph nodes and spatial transcriptome atlas remains unclear. In this study, by integrating single-cell RNA sequencing (scRNA-seq) and spatial transcriptomics, we investigate the transcriptional profiling of six surgically excised lymph node samples and the spatial organization of one positive lymph node. We identify the existence of osteoclast-like giant cells (OGC) which have high expressions of CD68 and CD163, the biomarkers of tumor-associated macrophages (TAMs). Through a spatially resolved transcriptomic method, we find that OGCs are scattered among metastatic breast cancer cells. In the lymph node microenvironment with breast cancer cell infiltration, TAMs are enriched in protumoral pathways including NF-κB signaling pathways and NOD-like receptor signaling pathways. Further subclustering demonstrates the potential differentiation trajectory in which macrophages develop from a state of active chemokine production to a state of active lymphocyte activation. This study is the first to integrate scRNA-seq and spatial transcriptomics in the tumor microenvironment of axillary lymph nodes, offering a systematic approach to delve into breast cancer lymph node metastasis.

## Introduction

Breast cancer has become the leading cause of death from cancer in women around the world, and axillary lymph nodes are the most common sites of metastasis [1]. Axillary lymph node dissection has been the standard treatment against lymph node metastasis, yet the postoperative recurrence rates remain higher than 10%, highlighting the significance to delve into the cellular and molecular mechanisms underlying this malignant process [2]. As a part of the immune system, lymph nodes are active in anti-tumor immunity, but as cancer cells invade and proliferate, the local immune response might fail, transforming positive lymph nodes into the start for further metastasis. It has been acknowledged that axillary lymph nodes are the first step in the process of distal metastasis initiation of breast cancers, and the presence of metastatic cancer cells in axillary lymph nodes is significantly associated with poorer clinical outcomes compared with patients with no lymph node metastasis [ 3, 4] . The prognostic significance as well as the role axillary lymph nodes play highlights the necessity to study the detailed mechanisms and molecular characteristics of the lymph node microenvironment, which could be altered as tumors progress [5]. Tumor microenvironment refers to the cellular and molecular components surrounding the tumor tissue, and an immunosuppressive microenvironment fosters the initiation of cancer cells to migrate distally, whereas a hospitable one hinders the progression of cancer [6].

With the constant emergence of new technologies, the molecular mechanisms of cancer biology have been explored in an unprecedentedly precise way. Single-cell sequencing enables the high-throughput analysis of tissues, making possible the annotation of newly-found small cellular populations, the accurate resolution of cell-cell interactions, and the comprehensive comparisons between tissues. In our previous study by using scRNA-seq we revealed the transcriptome characteristics of cancer cells during the malignant process of lymph node metastasis and further investigated the biological function of certain cancer cell subclusters, broadening the knowledge of how immune evasion is conducted by metastatic breast cancer cells [7]. Despite that lymph nodes are enriched in immune cells, cancer cells still survive and proliferate in this tumor microenvironment (TME), which inspires us to focus on TME components and explore whether these elements modulate a conducive microenvironment for metastatic tumor cells or are transformed into a more immunosuppressive state.

Although scRNA-seq provides advanced precision, transcriptomic sequencing can only be performed after tissue dissociation which damages spatial constructions, resulting in the loss of biomolecular information based on spatial relations. This limitation calls for spatial transcriptomics, a novel technique that has been applied to studies in the fields including cortical tissue, interverbal discs and several murine models [ 8– 11] . Through microarray barcodes, spatial transcriptomics maps the quantitative transcripts with spots from high-resolution imaging which is composed of 10‒200 cells [12]. Therefore, the integration of scRNA-seq and spatial transcriptomics makes up for the limitations of both techniques, showing promising value in a comprehensive and precise dissection of metastatic TME.

In this study, we included 6 surgically excised lymph nodes which were defined as positive lymph nodes by pathologists and dissolved the tissue for scRNA-seq. To investigate the detailed spatial characteristics of breast cancer lymph node metastases, one of the six positive lymph nodes was selected to perform spatial transcriptomics. Bioinformatics analysis which integrates single-cell transcriptomes with spatial characteristics unraveled the TME atlas of breast cancer lymph node metastasis, which provided a systematic overview for future research.

## Materials and Methods

### Tissue dissociation and preparation for single-cell sequencing

Six positive lymph nodes (PL1 to PL6) of six breast cancer patients were harvested after breast cancer surgeries from the First Affiliated Hospital of Nanjing Medical University (Nanjing, China). All experimental procedures were approved by the Ethics Committee of the First Affiliated Hospital of Nanjing Medical University and were conducted in compliance with the Helsinki Declaration. Pathologic examination was performed according to the current International Union against Cancer tumor-lymph node metastasis classification. GEXSCOPE™ Tissue Preservation Solution (Singleron Biotechnologies, Nanjing, China) was used to store fresh atherosclerotic plaque. The specimens were washed twice with Hanks balanced salt solution and cut into 1–2 mm pieces. Tissue pieces were then digested for 15 min with 2 ml of GEXSCOPE™ Tissue dissociation solution (Singleron Biotechnologies) at 37°C in a 15-mL centrifuge tube with uninterrupted agitation. After digestion, samples were filtered using 40-μm nylon sterile strainers and centrifuged at 350 *g* for 5 min. Then, the supernatant was removed and the pellet was resuspended in 1 mL PBS (HyClone, Logan, USA).

### Single-cell RNA sequencing (scRNA-seq)

Single-cell suspensions with 1×10 ^5^ cells/mL in PBS (HyClone) were prepared. The suspensions were then loaded onto microfluidic devices and scRNA-seq libraries were constructed according to the Singleron GEXSCOPE ^TM^ protocol using the GEXSCOPE™ Single-Cell RNA Library Kit (Singleron Biotechnologies) [13]. After quality checks, individual libraries were diluted to 4 nM and pooled for sequencing on an Illumina NovaSeq (Illumina, San Diego, USA) with 150 bp paired-end reads.

### Single cell RNA-seq data processing and quality control

Sequencing outputs were demultiplexed to convert BCL files to FASTQ format using bcl2fastq (Illumina), sequencing data were processed using the CeleScope1.1.7 pipeline ( https://gitee.com/singleron-rd/celescope; Singleron Biotechnologies). Adapters and poly A tails were trimmed (fastp V1) before aligning read two to GRCh38 using Ensemble v.92 gene annotation (fastp 2.5.3a and featureCounts 1.6.2). Reads with the same cell barcode, unique molecular identifier (UMI), and gene were grouped to calculate the number of UMIs per gene per cell. The UMI count tables of each cellular barcode were used for further analysis.

We use six samples for single-cell RNA-seq data (GSE180286, GSE188807). Six positive lymph nodes included in this study contained cells of the following number: 10546 (PL1), 3645 (PL2), 4935 (PL3), 4793 (PL4), 4011 (PL5) and 8035 (PL6). A total of 35,965 single cells were included in this research. The gene expression matrix was then processed and analyzed with Seurat v3.1.2 package. To filter out low-quality cells, we first removed cells which had less than 200 genes and higher than 98% gene number, less than 200 UMI and higher than 95% UMI number, and over 20% genes derived from the mitochondrial genome. More information on quality control is shown in Supplementary Table S1.

### Dimensionality reduction, clustering, cell-type labeling and visualization

Seurat v3.1.2 was used for dimensionality reduction, clustering and visualization. For each sample dataset, we used the filtered expression matrix to identify cell subsets. The filtered gene expression matrix was normalized using Seurat’s NormalizeData function, in which the number of UMIs of each gene is divided by the sum of the total UMIs per cell, multiplied by 10,000, and then transformed to logscale (ln (UMI-per-10000+1)). After data normalization, highly variable genes were identified and used for the following Principal component analysis (PCA). Harmony v0.1 was used to integrate samples and perform downstream analysis. Subsequently, clustering with 50 principal components and resolution 1.0 was performed by graph-based clustering and visualized using t-Distributed Stochastic Neighbor Embedding (t-SNE) or Uniform Manifold Approximation and Projection (UMAP) with Seurat functions RunTSNE and RunUMAP. For the clustering of T lymphocytes, the top 20 principal components (PCs) were selected with a resolution parameter of 0.3. For the clustering of myeloid cells, the top 20 PCs were selected with a resolution parameter of 0.5. For the clustering of Fibroblasts cells, the top 20 PCs were selected with a resolution parameter of 0.3. For the clustering of B cells, the top 20 PCs were selected with a resolution parameter of 0.5. Cell types were annotated with the SynEcoSys database (Singleron Biotechnologies).

The method returned cell clusters which were then visualized on UMAP space using the DimPlot command. Cell-type-specific canonical gene markers were used to label clusters which differentially express those markers. To accurately label individual clusters, the Wilcox test was performed to find differentially expressed genes for each cluster. The FindAllMarkers function in Seurat was used to get a list of differentially expressed genes for each cluster. Gene expression was visualized using FeaturePlot, DoHeatMap, and VlnPlot functions from Seuratv3.1.2.

### Sample preparation for 10× Visium spatial transcriptomics platform

The tissue sample of PL6 was immediately embedded in Optimal Cutting temperature Compound (OCT; 10× Genomics, Pleasanton, USA) and frozen in liquid-nitrogen-cooled isopentane bath, cut into 10 μm sections using Thermo Scientific CryoStar NX50 cryostat (Thermo Scientific, Waltham, USA), and mounted on 10× Visium slides, which were pre-cooled to −20°C.

### Preparation of 10× Visium spatial transcriptomics library

We used the 10× Genomics Spatial RNAseq Visium platform (10× Genomics, lnc.) for the spatial transcriptomics experiments of the PL6 sample (GSE189402). A 10× Genomics Visium Gene Expression slide has 4 capture areas, each with an array of 5000 circular spots containing printed DNA oligos for mRNA capture. These oligos on each spot have a PCR handle, unique spatial barcode, UMI, and a poly-dT-VN tail for capturing the 3′ end of mRNA molecules. Each spot with a unique spatial barcode is 55 μm in diameter and the center to center distance between the spots is 110 μm. One 55 μm spot captures mRNA from 10 to 20 cells depending on cell size and packing density which is variable across the tissue. These sections were fixed in pre-chilled methanol for 30 min, subject to H&E staining, and then imaged. H&E staining was used later by the 10× Genomics Cell Ranger software to detect the spots which were covered by the tissue. The optimal permeabilization time for 10 μm thick tissue sections was found to be 12 min using 10× Genomics Visium Tissue Optimization Kit. Spatially tagged cDNA libraries were built using the 10× Genomics Visium Spatial Gene Expression 3′ Library Construction V1 Kit. H&E-stained heart tissue sections were imaged using Zeiss PALM MicroBeam laser capture microdissection system and the images were stitched and processed using Fiji ImageJ software. cDNA libraries were sequenced on an Illumina NovaSeq output kits (Read 1=28, Read 2=120, Index 1=10, and Index 2=10). Fluidigm frames around the capture area on the Visium slide were aligned manually and spots covering the tissue were selected using Loop Browser 4.0.0 software (10× Genomics). Sequencing data were then aligned to the human reference genome using the Space Ranger 1.0.0 pipeline to derive a feature spot-barcode expression matrix (10× Genomics).

### Spatial RNA-seq data processing, integration and visualization

Spatial RNA-seq data from two 10× Visium capture areas with each area containing 5000 barcoded spots were first processed by Space Ranger analysis software. And the resultant matrix files were analyzed by Seurat v4.0.1 package according to the standardized pipeline. Another software, stLearn, based on the result of Seurat analysis, absorbed the spatial distance, tissue morphology and gene expression from the raw count of the data and images. After filtering, normalization and log1p, stLearn extracted high-level features from the tile images. Then, stLearn ran PCA for gene expression data and applied stSME to normalize log-transformed data. Finally, several populations were clustered by Louvain on stSME normalized data.

Spatial transcriptomics deconvolution was started with Seurat label transferring. Data with cell types annotated from scRNA-seq and Spatial RNA-seq data did the re-normalization and PCA by Seurat using SCTransform and RunPCA. Then, FindTransferAnchors was used to find anchors for integration with the two datasets. The underlying composition of cell types was predicted to deconvolute each of the spatial voxels by TransferData method. In this step, in order to avoid noise and cells with low confidence, cells with a prediction ratio greater than 70% will be kept. At the same time, if the largest predicted ratio of the cell type of a cell is between 50% and 70%, and the second ratio is less than 20%, cells will also be retained for future analysis. Through the above steps, a table containing the proportion of cell type contained in each cell from Spatial RNAseq data is available. This table was passed to stLearn, and used to do the visualization and classify the cell type composition in each cluster. The degree of colocalization of cell-type pairs within spots in data was visualized by using this table as well which counted the number of each cell type that existed in each cell.

### Pseudotime and gene regulon analysis

We explored the cell states and cell transitions of macrophages in lymph nodes with breast cancer metastasis by inferring the state trajectories using Monocle2. The pySCENIC (1.10.0) was used with its command-line implementation. Pyscenic grn command was used with grnboos2 method and default options and a fixed seed to derive co-expression modules between transcription factors and potential targets. The motifs database for Homo sapiens was downloaded from the website https://pyscenic.readthedocs.io/en/latest/. Only positive regulons (i.e., those with a positive correlation between the TF and its targets) were kept for downstream analysis. AUCell scores (regulon activities) in each cell were computed with pycenic aucell command (default options).

### Differential gene expression analysis and enrichment analysis

Differentially expressed genes in a given cell type compared with all other cell types were determined with the FindAllMarkers function from the Seurat package (Wilcoxon rank-sum test, *P* values adjusted for multiple testing using the Bonferroni correction). To compute differentially expressed genes, all genes expressed in at least 10% of cells in either of the two populations were compared and the expression difference on a natural log scale was at least 0.25. Differentially expressed genes were filtered by |fold change|>0.25 and FDR<0.05. For the differentially expressed genes of different cell types, genes with *P* value<0.05 and |logFC|>0.25 were selected for enrichment analysis. The up-regulated genes and down-regulated genes were analyzed separately. The GO and KEGG enrichment analyses were performed by clusterProfiler (3.16.1).

### Cell-cell interaction analysis

To analyze cell-cell interactions between different cell types, CellPhoneDB (Vento-Tormo *et al*., 2018) was used to identify significant ligand-receptor pairs [14]. Statistical analysis of CellphoneDB was used for data analysis, and default parameters were used.

### Immunofluorescence assay

Immunofluorescence staining was used to verify the expressions of CD68 and CD163. The tissue sections of PL6 were deparaffinized and rehydrated, followed by antigen retrieval. After being blocked for 1 h in 3% bovine serum albumin (BSA) at 37°C, the tissue sections were incubated overnight at 4°C with the following primary antibodies: rabbit anti-CD68 antibody (1:100; Abcam, Cambridge, UK) and mouse anti-CD163 antibody (1:100; Abcam). Subsequently, sections were incubated with the secondary antibodies for 1 h at 37°C, followed by counterstaining with DAPI. The tissue sections were then observed and photographed under the inverted microscope.

### Data and materials availability

We selected scRNA-seq data of 4 positive lymph nodes (PL1 to PL4) from our published study GSE180286. To better understand the microenvironment of positive lymph nodes, the scRNA-seq data of two more positive lymph nodes have been deposited in the GEO database under accession code GSE188807. Additional data related to this paper may be requested from the authors. The spatial transcriptomics data of the PL6 sample have been deposited in the GEO database under accession code GSE189402.

## Results

### Overview of lymph nodes with breast cancer metastases

**Figure 1:**
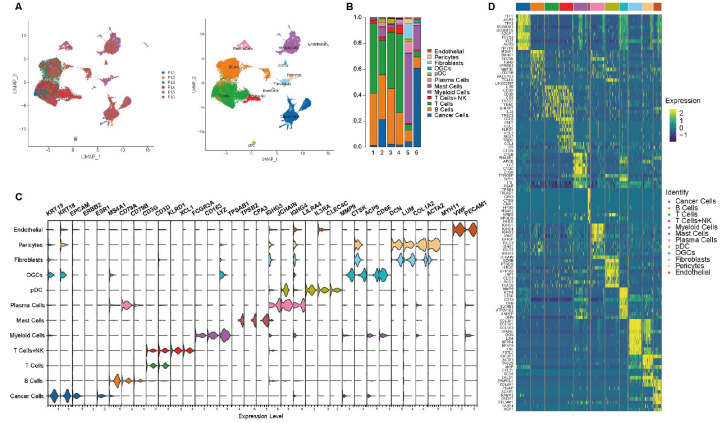
Figure 1 Overview of lymph nodes with breast cancer metastases (A) UMAP plots of 35,965 single cells from six positive lymph nodes, colored according to sample origins and cell types. (B) Cellular composition of samples according to sample origins. (C) Violin plot showing biomarkers of different cell types. (D) Heatmap showing differentiated expressed genes of cell types. PL, positive lymph node. OGC, osteoblast-like giant cell. pDC, peripheral dendritic cells. NK, natural killer cells.

In our previous study, we employed scRNA-seq in the research on the differentiation of cancer cells between metastases and primary tumors (GSE180286) [15]. To better understand the microenvironment of positive lymph nodes, we included two more positive axillary lymph nodes, making up six lymph nodes with breast cancer cell infiltration ( Figure 1A). All six samples were confirmed to be positive lymph nodes (PLs) with breast cancer metastases by scRNA-seq. The scRNA-seq data consisted of 35,965 cells from six positive axillary lymph nodes. After quality control and batch-effect removal, unique transcripts were normalized. Subsequently, a total of twelve distinct cell types were grouped according to their transcriptome profiles, and uniform manifold approximation and projection (UMAP) plots were employed for visualization ( Figure 1A). Next, we analyzed the cellular composition of the respective lymph nodes and found that PL6 was the lymph node most invaded by breast cancer cells ( Figure 1B). To infer the identification of single cells, transcriptome profiles were referred to as the canonical biomarkers for annotation of distinct cell types ( Figure 1C). Heatmap analysis of the top differentially expressed genes (DEGs) delineated the signatures of each cell type among the six positive lymph nodes, with cancer cells highly expressing genes including *TFF1*, *AGR2*, *TFF3*, and myeloid cells highly expressing genes including *C1QB*, *RNASE1* and *APOE* ( Figure 1D). Noticeably, we identified a group of cells with osteoclast biomarkers and high expression of genes including *MMP9*, *CST3* and *CD68* [16]. According to previous studies on invasive breast carcinoma [ 17‒ 19] , we further grouped this cell type to be osteoclastic giant cells (OGCs).

### Transcriptome profiles of lymphocytes in metastatic lymph nodes

**Figure 2:**
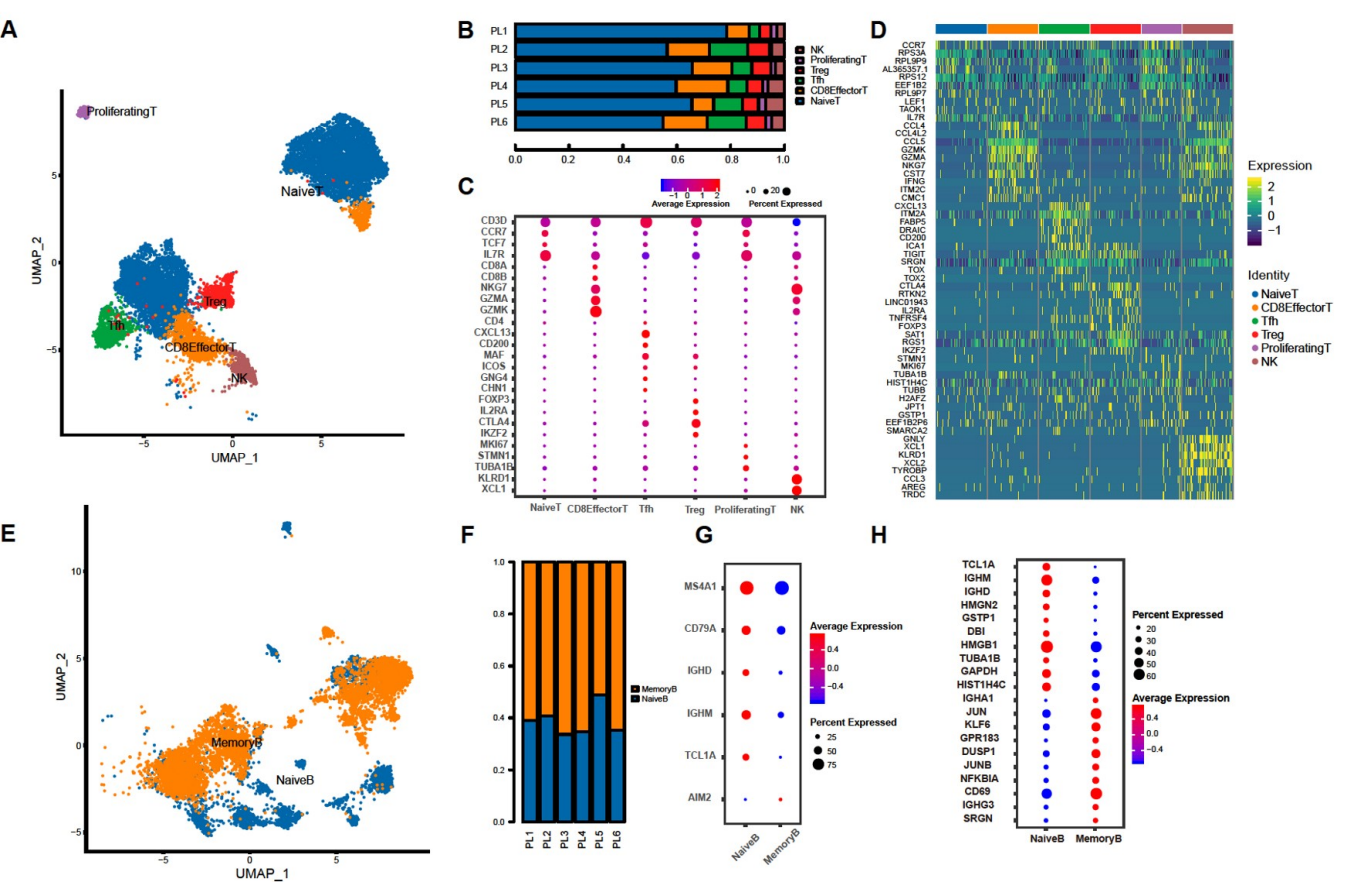
Figure 2 Transcriptome profiles of lymphocytes in metastatic lymph nodes (A) UMAP of T lymphocytes from six positive lymph nodes, colored according to cell types. (B) T lymphocytes composition in different samples. (C) Biomarkers of T lymphocyte subclusters. (D) Heatmap showing differentiated expressed genes of T lymphocytes. (E) UMAP of B lymphocytes from six positive lymph nodes, colored according to cell types. (F) B lymphocytes composition in different samples. (G) Biomarkers of B lymphocyte subclusters. (H) Differentiated expressed genes of B lymphocytes. Treg, regulatory T cells. Tfh, T follicular helper cells.

Among all the twelve cell types identified from 35,965 cells, T cells took the largest part ( Figure 1C). Reclustering identified six T cell subclusters, including natural killer T cells (NK, NKG7+, KLRD1+, XCL1+, *etc*.), proliferating T cells (CD3D+, IL7R+, CCR7+, *etc*.), regulatory T cells (Treg, FOXP3+, CTLA4+, IL2RA+, *etc*.), follicular helper T cells (Tfh, CXCL13+, CD3D+, MAF+, *etc*.), CD8 effector T cells (CD8A+, CD8B+, GZMK+, *etc*.), and naïve T cells (IL7R+, CCR7+, TCF7+, *etc*.) which accounted for the highest proportion of the total ( Figure 2A‒C). Cells from all T cell subclusters were detected in positive lymph nodes from all patients. DEGs of distinct T cell clusters were identified ( Figure 2D).

Different from blood NK cells that express cytotoxic perforin and granzyme genes, in the TME of metastatic lymph nodes, NK cells possess high expressions of chemokines, such as XCL1, XCL2, CCL3, and CCL5 which contribute to the recruitment of myeloid cells [20]. However, NK cells in the metastatic TME specifically express CD96, a negative regulator of NK cell cytotoxicity [21]. Moreover, as a contributor of functional switch into a pro-tumoral NK cell state, KLRG1 is highly expressed in the TME of breast cancer lymph node metastases [22]. FOXP3 and BATF validate the identification of Treg cells which is enriched in immune checkpoint molecules including CLTA4 and TIGIT, co-stimulatory molecules such as TNFRSF18, and transcriptional regulators including MAF and IKZF2 [ 12, 23, 24] . Treg cells in metastatic TME also highly express CD69 which is a biomarker of T cell activation and IL2RA which encoded the high-affinity receptor of interleukin 2 [25]. Similar to Treg cells, the CXCL13 ^+^ Tfh cell cluster was also detected to have differential overexpressions of co-inhibitory molecules including TIGIT and PDCD1. BTLA which took part in an inhibitory cell-cell interaction was also highly expressed in Tfh cells [26]. CD8 effector T cells in lymph nodes have specifically high expressions of genes including *CCL4*, *CCL5*, *IFNG*, *HLADR* and *CD69*, indicating the pro-inflammatory phenotype of CD8 effector cells in positive lymph nodes; meanwhile, the elevated expression of cytolytic biomarker GZMA also suggested its potential of immunotherapy trials [ 27‒ 29] . In addition to inflammation-related genes, this CD8 lymphocyte subcluster highly expressed HLADR isotype and CD69 which were previously reported to promote cancer apoptosis [ 30, 31] . Naive T cells took the largest part in positive lymph nodes from all six patients, yet differential overexpressions of the co-inhibitory receptors CTLA4 and TIGIT were detected, suggesting the possible exhaustion of T cells in the TME of axillary lymph nodes [32].

B cells are clustered into naïve B cells and memory B cells according to the respective biomarkers ( Figure 2E‒G). As a biomarker of cell metabolism, MKI67 is significantly overexpressed in the naïve B cells while underexpressed in memory B cells ( Figure 2H) [33]. Immunoglobin-encoding genes including *IGHA* and *IGHG* were also highly expressed in both subclusters. The oncogenic transcription factor genes *KLF6* and *MARCKS* which encodes a protein that sequesters PIP2 were highly expressed in both naïve and memory B cells, indicating an increased proliferation and motility of immune cells in positive lymph nodes [ 34– 36] . Generally, the clustering of B cells did not show obvious differences across subclusters as T cells did.

### Transcriptome profiles of myeloid cells in metastatic lymph nodes

**Figure 3:**
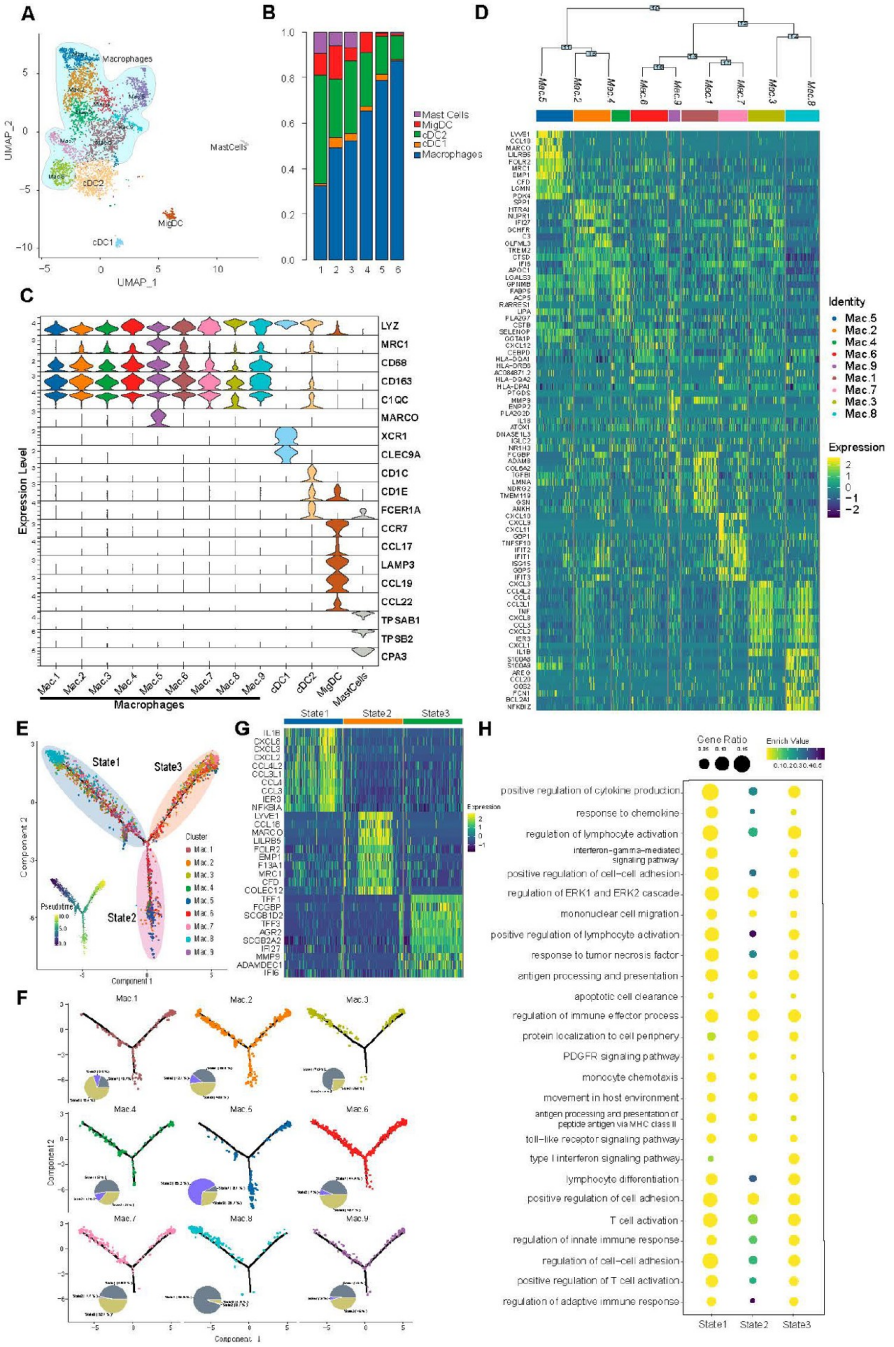
Figure 3 Transcriptome profiles of myeloid cells in metastatic lymph nodes (A) UMAP of myeloid cells from six positive lymph nodes, colored according to cell types. (B) Cellular composition of samples according to sample origins. (C) Violin plot showing biomarkers of different macrophage subclusters. (D) Heatmap showing differentiated expressed genes of myeloid cell types, and differentiation tree showing the transcriptomic similarities of macrophage subclusters. (E) Trajectory analysis of three states of macrophage subclusters. (F) Composition of macrophage subclusters at different cell states. (G) Differentiated expressed genes of three states of macrophage differentiation. (H) Enrichment analysis of three states of macrophage differentiation. Mac, macrophage. Mig DC, migratory dendritic cells. cDC, classical dendritic cells.

In terms of myeloid immune cells, mast cells took the smallest part in positive lymph nodes according to the cellular composition of the lymph node TME ( Figure 3A,B). Though best known in allergy and anaphylaxis, the TME mast cells highly express immunosuppressive IL6R and ferroptosis-related signatures including PEBP1 and NCOA4 [ 37, 38] . S100A8 and S100A9 are also highly expressed in TME mast cells which promote tumor proliferation and metastasis and indicate poor outcomes according to previous research [39]. As antigen-presenting cells, the dendritic cells have been crucial in antitumor response. scRNA-seq clusteres dendritic cells (with marker genes *ID2*, *CLEC4*, *IRF4*, and *CD83*) into three subclusters, i.e., cDC1, cDC2 and migDC, and it is known that cDC1 presents antigens to CD8 ^+^ T cells while cDC2 presents antigens to CD4 ^+^ T cells ( Figure 3C) [40]. Interferon receptors (IFNGR1 and IFNGR2) are expressed in the three DC clusters.

Notably, the TME macrophages were identified by canonical marker genes including *CD14*, *CD68*, *FCGR3A* and were clustered into nine distinct subclusters (Mac.1-9) across all six positive lymph nodes ( Figure 3D) [12]. Generally, macrophages are divided into two main subtypes, the pro-inflammatory M1 subtype and the pro-tumoral M2 subtype which is also known as tumor-associated macrophages (TAM) [ 41, 42] . Interestingly, the potential homology of certain macrophage clusters was found, prompting us to do trajectory analysis to study the dynamic differentiation. As a result, we detected three states across the development of these nine macrophage subclusters ( Figure 3E). The majority of macrophage subclusters are enriched in state1 and state3, Mac.5 is mostly enriched in state2 and Mac.8 is mostly enriched in state1 ( Figure 3F). DEGs and enriched pathways were identified across cell clusters and developmental states to analyze heterogeneous phenotypes, showing differential biological hallmarks of gene transcription ( Figure 3G,H). State1 is characterized by cytokine production and response to chemokines, state2 is characterized by processing of exogenous antigens and monocyte chemotaxis, and state3 is characterized by cell-cell adhesion and lymphocyte differentiation ( Figure 3H).

**Figure 4:**
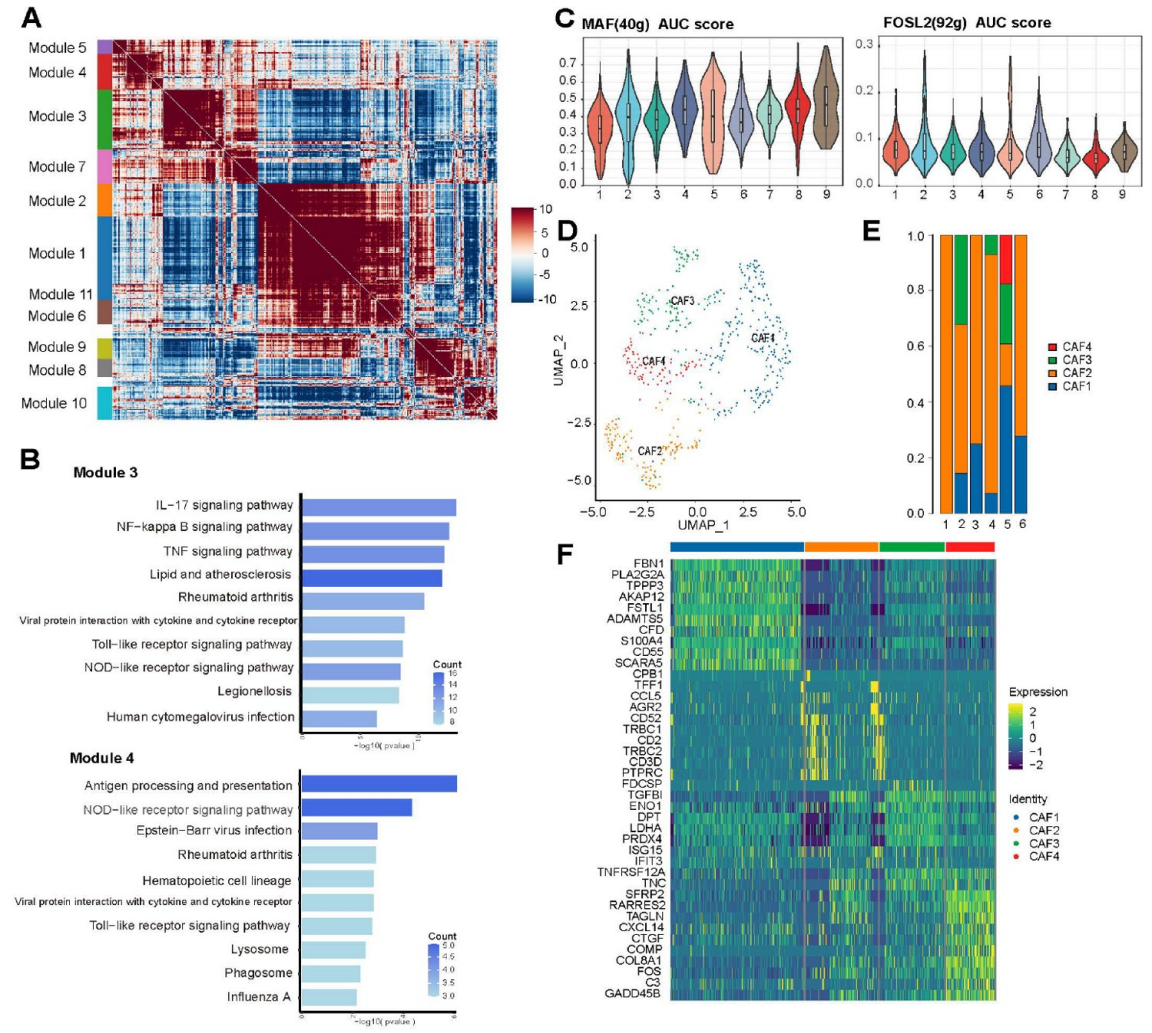
Figure 4 Transcriptome characteristics of macrophage subclusters (A) Gene modules delineated the transcriptome characteristics of macrophages at the gene collection level. The red and blue part of the heatmap referred to macrophages with high or low expression of genes which were lined up together according to similarities. (B) KEGG enrichment analysis of representative modules identified in A. (C) Bioinformatics analysis based on pySCENIC delineated the characterization of macrophage subclusters from the transcription perspective, identifying the transcription factors together with the corresponding targeted genes among macrophage subclusters. Here, the expression of representative transcription factors in macrophage subclusters is shown. (D) UMAP of CAFs from positive lymph nodes. (E) Cellular composition of CAFs in six positive lymph nodes. (F) Differentially expressed genes of CAF subclusters. AUC, area under curve. CAF, cancer-associated fibroblasts.

Next, we investigated the characteristics of macrophages at the level of the gene set. Gene set refers to a set of genes with inner similarities. This analysis was performed by Hotspot, a gene function set algorithm based on the graph. Bioinformatics analysis sorted out eleven gene sets (Module 1-11) according to transcriptome profiles of macrophages ( Figure 4A). The correspondence between modules and cell clusterization showed that Module 3 is most enriched in Mac.1, Mac.5 and Mac.8, and Module 4 is highly enriched in almost all macrophage subclusters. Further analysis revealed that Module 3 is enriched in IL-17, NF-κB, TNF, and Toll-like receptor signaling pathways, and Module 4 is enriched in antigen processing and presentation, NOD-like receptor and Toll-like receptor signaling pathways ( Figure 4B). With a general understanding of macrophage subclusters, we further analyzed the transcription factors mediating the DEGs of macrophages. Based on transcriptome profiles, we detected high expression of transcription factors including MAF and FOSL2 ( Figure 4C). It was reported in T cells that C5AR1 elevates MAF expression and initiates Tfh cell differentiation [43], however in this research, we identified in TME macrophage the correlation between MAF as a transcription factor and C5AR1 as a DEG. We also identified co-expression of MAF and CCR5 in macrophages, which was reported to be related to PD-1 expression by immune cells [44]. We also detected co-expression of IRF7 and TIM-3 which is a biomarker of immune cell dysfunction [45]. The current analysis delineated the transcription profiles of TME macrophages from levels of differential expression, differentiation trajectory, gene sets, and transcription regulation.

### Transcriptome characteristics of other cellular components in positive lymph nodes

The tumor microenvironment consists of not only malignancy cells and immune cells, but also stromal cells in which cancer-associated fibroblasts (CAFs), fibroblasts surrounded cancer cells in tumor tissues, take a great part [46]. The main biological functions of CAFs include matrix deposition and remodeling, direct and indirect cellular interactions [47]. Moreover, it was reported that CAFs facilitate the interaction with cancer cells via EMT and the TGF/Smad signaling pathway, further contributing to tumor growth [46]. In this research, CAFs were identified in positive lymph nodes with breast cancer cell metastasis and were distinguished into four subclusters ( Figure 4D,E).

**Figure 5:**
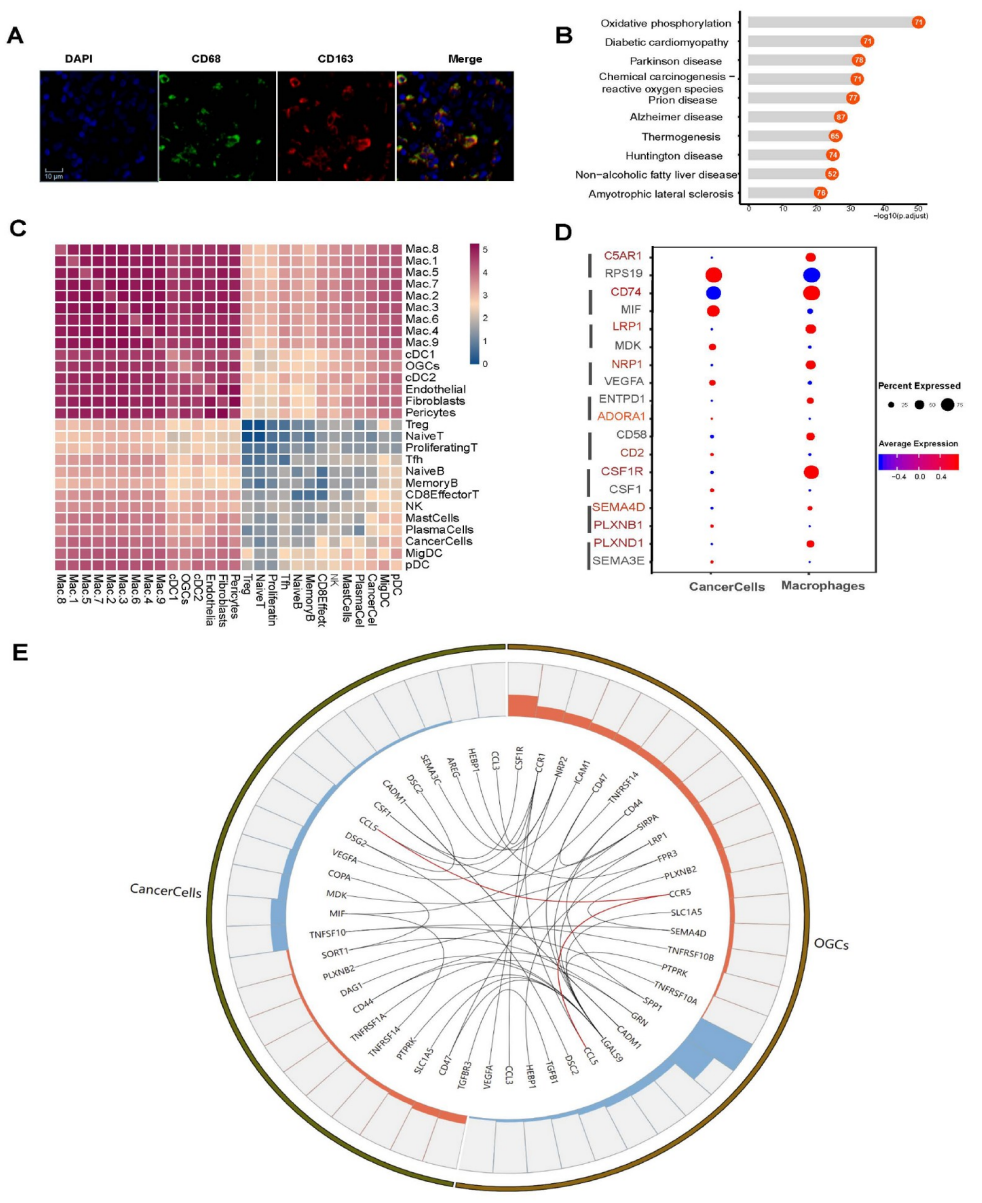
Figure 5 Transcriptome characteristics of osteoclast-like giant cells and cancer-immune interaction in lymph node TME (A) Immunofluorescence staining of CD68 and CD163 in PL6. (B) Enrichment analysis of DEGs in OGCs. (C) Heatmap showing the incidence of ligand-receptor pairs with different frequencies between different cell clusters. Numbers to the bar refer to the number of specimens that the interaction was found (for example, “5” refers to interactions found among five positive lymph nodes). (D) Representative ligand-receptor interactions between macrophage and cancer cells. (E) Representative ligand-receptor interactions between OGCs and cancer cells.

FBN1 is highly expressed in this cluster, indicating activity in an extracellular matrix organization and EMT process [48]; CAF in metastatic microenvironment highly expresses CCL5 which promotes the migration of tumor cells [49]; while high expression of tumor-suppressive genes such as *TFF1* [50] was also detected ( Figure 4F). We also identified a cell cluster with high expressions of osteoclast biomarkers including ACP5, CTSK, MMP9 and CD68 ( Supplementary Figure S1). Tumor with osteoclast-like giant cells was reported to be rare among breast cancers (<2%) [19]. With high CD68 expression and CD163L1, this cell cluster have similar characteristics with M2-macrophages ( Supplementary Figure S1) [19]. Immunofluorescence staining was performed to examine the expressions of macrophage biomarkers ( Figure 5A). All OGCs were derived from PL6, a LuminalB subtype with 90% ER and PR positivity and 35% Ki-67. GO enrichment showed the biological functions of overexpression genes in OGC, and oxidative phosphorylation is the highest-enriched pathway ( Figure 5B).

### Cancer-TME interaction in lymph node TME

Single-cell transcriptome atlas of positive lymph nodes revealed the ligand-receptor (L-R) pairs identified among cell clusters. Next, we tried to figure out the frequencies of L-R pairs among all six positive lymph nodes, identifying high frequency L-R pairs. Figure 5C showed the occurrence of pairs at different frequencies among clusters identified previously, leading us to focus on interactions between macrophages and cancer cells, endothelial cells and cancer cells, as well as OGC and cancer cells.

Macrophages took a great part in positive lymph nodes ( Figure 3B), and by scoring all macrophage subclusters on transcriptional characteristics of M1 (CD86, CCL19, CCL15, TNF) and M2 (CD163, CCL18, CCL13, MRC1) macrophages, we found that TME macrophages of lymph node metastases inclined towards M2 subtype, which is known as TAMs, and TAMs were recognized to be a prognostic factor to poor outcomes [ 19, 27, 42, 51, 52] . Single-cell transcriptome analysis demonstrated the representative interactions between macrophages and cancer cells ( Figure 5C,D). The pro-tumoral pair MIF-CD74 between metastatic cancer cells and several types of immune cells was detected in five out of six positive lymph nodes [ 53, 54] . RPS19, one of the DEGs in metastatic breast cancer cells, is an immunosuppressive molecule expressed by metastatic breast cancer cells, and its interaction with C5AR1 of macrophages was also identified in the metastases of five lymph nodes [55]. Breast cancer cells expressing CD47, an immune checkpoint molecule that interacts with macrophages expressing LGALS9, were reported to correlate with poor clinical outcomes [56]. This interaction, identified in five out of six positive lymph nodes, is defined to be a high-frequency interaction as well. CD74-APP interaction was reported to ameliorate beta-amyloid production in Alzheimer’s disease, but in five out of six positive lymph nodes, we detected this interaction between TME macrophages and metastatic breast cancer cells [57].

Despite a smaller percentage of cells compared with immune cells, endothelial cells are not only a part of the connective tissue but also enable the blood supply of the tumor. Moreover, Brown *et al*. [58] reported that lymph node vessels could serve as an exit route for cancer cells to disseminate systemically. ACKR1 is a molecule expressed by cells lining high endothelium venules, contributing to the pro-migratory ability of endothelial cells [59]. The CCL5-ACKR1 interaction between metastatic cancer cells and endothelial cells is also one of the high-frequency interactions occurring in at least five out of six lymph nodes. Based on transcriptome analysis and ligand-receptor interactions, we found that CCR5-CCL5, LGALS9-PTPRK could also take part in the OGC-cancer cell interaction ( Figure 5E).

### Integrative analysis of spatial and single-cell transcriptome analysis

**Figure 6:**
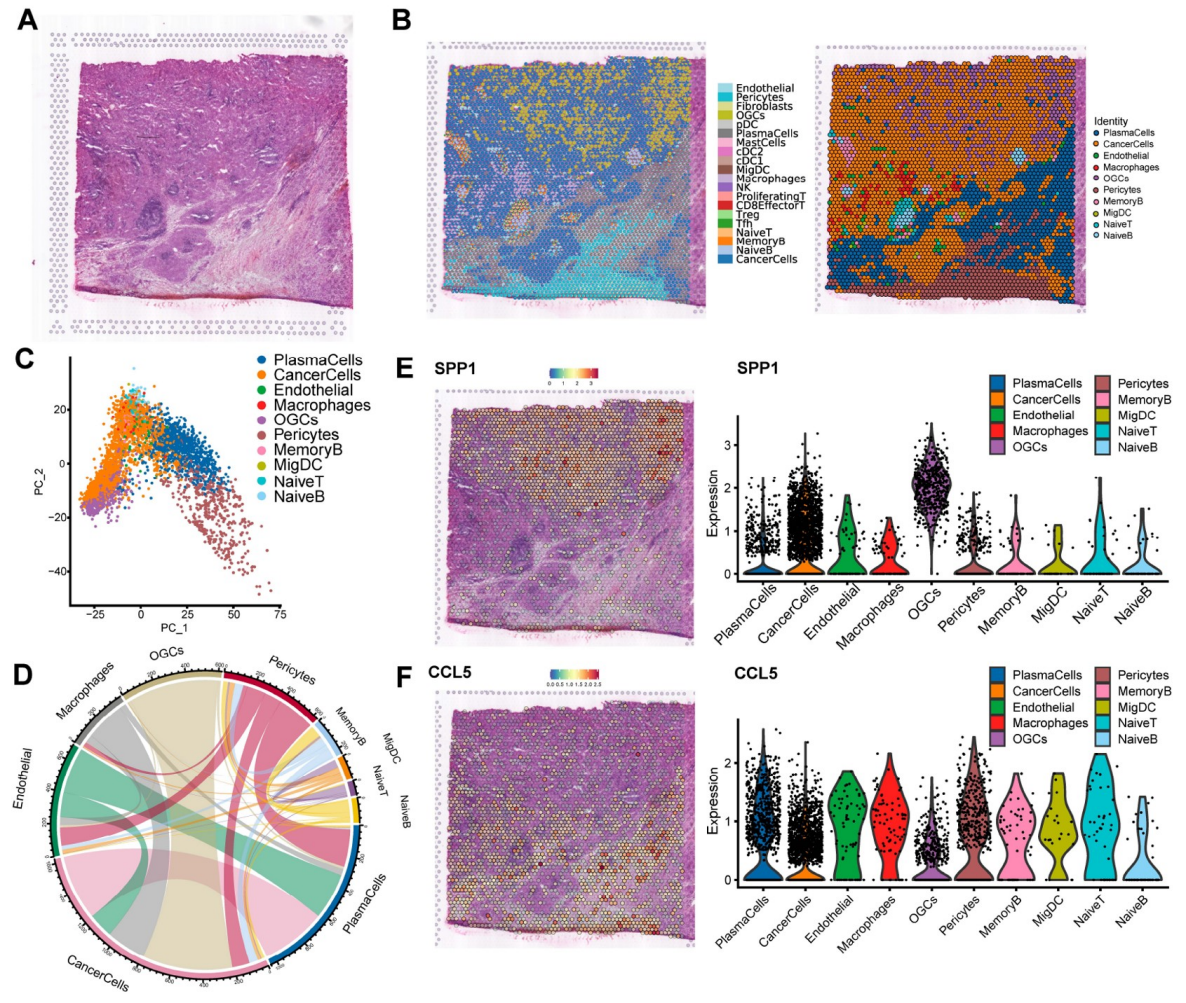
Figure 6 Integrative analysis of spatial and single-cell transcriptome analysis (A) H&E staining of PL6. (B) Annotation of plots from PL6. (C) UMAP of cell clusters from PL6. (D) Spatial localization relationship of cells indicating cellular interactions. (E,F) Representatives of differentially expressed genes in PL6 on spatial positioning and cell types.

For a better understanding of the transcriptomic landscape of breast cancer metastasis, spatial transcriptomics from 10× Genomics Visium was employed to investigate the correlation between transcriptional and spatial characteristics. We then performed spatial transcriptomics on PL6 for further exploration ( Figure 6A).

Since there is more than one cell in each spot, we decomposed and annotated each spot based on cell type biomarkers and deconvolution analysis ( Figure 6B,C). The majority of PL6 was composed of metastatic breast cancer cells with plasma cells and macrophage infiltration and scattered OGCs, and the original lymph node sinuses were disrupted by the metastatic cancer cells. By exploring the transcriptome characteristics for cell clusters in the positive lymph node with breast cancer metastasis, we identified that metastatic cancer cells distribute in most areas of the positive lymph node and the top differentially expressed genes included *NPY1R*, *AGR2*, *XBP1*, *RPL13*, *AGR3*, *IL6ST*, *FTL*, *COL6A2*, and *GATA3* ( *P*<0.0000). NPY1R, a novel peripheral blood marker, is upregulated in breast cancer, promoting proliferation and migration, and is positively correlated with metastatic diseases and highly expressed in circulating tumor cells among breast cancer patients [ 60, 61] . AGR2 was detected in disseminated tumor cells by several studies with its expression associated with Luminal subtype and breast cancer metastasis [62]. XBP1 is involved in chemoresistance and progression in TNBC, and XBP1 depletion impairs angiogenesis and promotes sensitivity to chemotherapy in HER2-positive breast cancer [63]. The high XBP1 expression in metastatic cancer cells from PL6 indicates a relationship between XBP1 expression and Luminal B breast cancer metastasis. OGCs are scattered among cancer cells, providing spatial contact for cellular interactions between OGCs and cancer cells ( Figure 6D). Figure 6D shows the spatial orientation of various cell clusters, the linkage between cell clusters illustrates the spatial proximity of cells, suggesting that OGCs are located adjacent to cancer cells. Likewise, the spatial adjacency of macrophages and cancer cells indicates the dense interaction between the two cell clusters.

We next explored the gene expression profiles of cell clusters from PL6 ( Figure 6E,F). High SPP1 expression was observed in TAMs and tumor tissues with advanced TNM stages in lung carcinomas [63]. According to this spatial transcriptomics analysis, we identified significant SPP1 expression in OGCs together with cancer cells ( Figure 6E). In addition, EMT biomarkers CDH1 and VIM were highly expressed in cancer cells, consistent with previous reports showing that TAM promoted EMT progress of cancers (lung cancer, colorectal cancer, breast cancer and ovarian cancer) by SPP1 [64]. As a key mediator of immune recruitment in tumor tissues, CCL5 is highly expressed in plasma cells and cancer cells ( Figure 6F) [65]. CCL5 expression by immune cells was reported to be a contributor to the EMT process of locally advanced breast cancer [66].

## Discussion

In this study, we revealed the transcriptome atlas of the microenvironment in positive lymph nodes. The integrative analysis of single-cell transcriptome profile and spatial transcriptomics portraited the characteristics of TME cells and the tumor-immune interactions in breast cancer lymph node metastasis. We further studied the cellular composition of TME, identified the DEGs and enriched functions of distinct cell types, demonstrated the interactions between cell types, and delineated a new subcluster of macrophages promoting immunosuppression during lymph node metastasis. Additionally, one of six positive lymph nodes was detected with OGCs whose occurrence rate was lower than 2% in all breast cancer specimens [ 18, 19] .

Single-cell transcriptome analysis revealed the characteristics of TME cell types in axillary lymph nodes with metastatic breast cancer cells. Significant expressions of co-inhibitory receptors including PDCD-1, TIGIT and TIM3 were identified in immune cells, and other tumor immunity-related genes were also highly expressed. It was reported that for various solid tumor models, co-inhibition of CD96 and PD-1 impedes lung metastases, increasing the production of local IFNγ and infiltration of lymphocytes [21]. Our findings revealed a significant expression of CD96 in TME NK cells in lymph nodes, indicating that CD96 could also be a potential target for controlling breast cancer metastasis in the early stage. We identified significant FOXP3 and BATF expressions in TME Treg cells. FOXP family expression has dual effects on cancer prognosis. For example, FOXP1 protein overexpression is associated with poor prognosis in several hematological diseases, while serving as a tumor suppressor in breast cancers; FOXP3 also indicates a better prognosis for breast cancers [23]. Tfh cells in metastatic TME highly express BTLA which was reported to be an inhibitory molecule on lymphocytes, hindering lymphocyte development and suppressing tumor immunity [26]. This single-cell transcriptome analysis revealed elevated expressions of CCL4 and CCL5 in CD8 effector cells of lymph node metastases. However, CCL4 and CCL5 are indicators of pro-inflammatory phenotypes and associated with better antitumor activity in resectable and metastatic PDAC [28]. For B lymphocytes, the biological functions such as immunoglobulin production and proliferation are active in metastatic TME, which is consistent with previous research on tumor immune response [ 34– 36] . IL-6 was reported to suppress the anti-tumor functions of T cells, which therefore impedes the effects of immunotherapy in patients [38]. Research on protein basis showed that high S100A/S100A9 expression could serve as an indicator for clinical outcomes in lung cancer, and this single-cell transcriptome study demonstrated significant expressions of these proteins in breast cancer lymph node metastases [39]. We clustered macrophages into nine subclusters by transcriptome characteristics, and all nine subclusters show an inclination to M2 macrophage biomarkers rather than M1, which suggests that after being invaded by metastatic cancer cells, macrophages undergo polarization into TAMs in lymph nodes [ 19, 42, 51] . In this study, we summarized the characteristics of RNA profiles in TME cells, by referring to reports in other fields, and revealed the biological functions of the DEGs, which enabled further validation of more detailed mechanisms.

When studying the cellular components of positive lymph nodes, we identified OGC which is rare among breast cancer reports. The primary tumor of PL6 was examined as T2N3c with 90% expression of ER, PR and 35% expression of Ki67. Breast cancer with OGCs was reported in less than 2% of all breast cancers, and correlated with estrogen receptor-positive subtype and low Ki-67 (<10%) [18], in line with our sample except Ki-67 which is a marker of intense metabolism. We identified the transcriptomics characteristics of OGCs including SPP1, ACP5, and CTSK. Previous research on melanoma showed that SPP1 is a pro-tumoral driver and prognostic factor in melanoma, with bromodomain and extra-terminal domain (BET) inhibitor being a therapeutic agent targeting this protein [67]. ACP5 was reported as a lysosomal acid phosphatase important to protein processing in osteoclasts, and our findings showed that *ACP5* is a potential marker gene in OGC [68]. Research on colorectal cancer revealed that CTSK is a secretory protein related to tumor metastasis and contributes to poor prognosis *in vivo* [69]. It is the first single-cell RNA sequencing on positive lymph nodes that delineates the transcriptomics profile of OGC in breast cancer.

To survive in lymph nodes where immune cells are enriched, the metastatic breast cancer cells exert immunosuppressive effects on TME cells. Intense interactions take place among macrophage subclusters, between macrophages and cancer cells, indicating immune escape in metastatic lymph nodes. We identified a rare cell cluster OGC which expresses M2-macrophage biomarkers. Immunohistochemical (IHC) analysis of 92 breast cancer patients in Egypt reported higher expression of NPY1R in breast cancer tissues compared with non-neoplastic tissues and a positive correlation between NPY1R protein expression and presence of metastasis ( *P*<0.001), clinical stages ( *P*<0.0003), and estrogen receptor expression ( *P*=0.004) [61]. In our research, PL6 was harvested from the surgical specimen of a female patient with LuminalB subtype, diagnosed as T2N3c with multiple vascular invasions by pathological examination. In metastatic breast cancer, we identified high expression of AGR2, the monoclonal antibody of which was reported to inhibit lung cancer growth and metastasis via the p53 pathway [70].

In summary, this integrative analysis of scRNA-seq and spatial transcriptomics revealed a comprehensive atlas of TME in lymph nodes with breast cancer cell invasion, delineated the characteristics of various cell types, demonstrated the tumor-immune interactions in positive lymph nodes, and identified a small cellular population. Admittedly, only the sixth patient sample was analyzed in this study with both scRNA-seq and spatial transcriptomics. Larger sample sizes and well-designed functional experiments are needed to validate our findings. This report serves as a primary reference for future studies on breast cancer lymph node metastasis and immune evasion by metastatic cancer cells.

## Supplementary Data

Supplementary data is available at *Acta Biochimica et Biophysica Sinica* online.
